# Breastfeeding practices and challenges in women with breast implants in Saudi Arabia: a descriptive study

**DOI:** 10.3389/fgwh.2025.1647351

**Published:** 2025-09-02

**Authors:** Abeer Orabi

**Affiliations:** 1 College of Nursing, King Saud bin Abdulaziz University for Health Sciences, Jeddah, Saudi Arabia; 2 King Abdullah International Medical Research Center, Jeddah, Saudi Arabia

**Keywords:** breast augmentation, breastfeeding, breast implants, cosmetic surgery, lactation

## Abstract

**Introduction:**

Breast augmentation is increasingly common in Saudi Arabia, yet little is known about its effect on breastfeeding. Given cultural norms favoring breastfeeding, understanding lactation outcomes in women with breast implants is vital for informed clinical counseling. This study aims to describe breastfeeding practices and challenges among women in Saudi Arabia with breast implants.

**Methods:**

A cross-sectional, descriptive study was conducted using an online survey distributed via social media. The sample included 240 women with a history of breast implants who had given birth in the past five years and were currently breastfeeding. The survey collected data on demographics, surgical history, feeding practices, and perceived breastfeeding challenges. Data were analyzed using descriptive and inferential statistics to assess associations with breastfeeding experiences.

**Results:**

Participants had a mean age of 28.5 years; 50.4% initiated breastfeeding immediately postpartum and 46.3% practiced exclusive breastfeeding. Commonly reported challenges included breastfeeding-related pain (55.0%), perceived insufficient milk supply (48.3%), and nipple or breast issues (48.3%). Supplementary feeding was reported by 49.6% of respondents. Sociodemographic factors such as maternal age, marital status, and number of children were significantly associated with breastfeeding duration (*p* = 0.041, *p* = 0.027 and *p* = 0.026, respectively). Regression analysis revealed that married women had 62% lower odds of breastfeeding beyond six months compared to divorced women (OR=0.38, 95% CI: 0.17–0.87, *p* = 0.022).

**Discussion:**

Breastfeeding is feasible for many women with breast implants in Saudi Arabia; however, some face notable challenges. These findings highlight the need for individualized preoperative counseling and specialized postpartum lactation support to improve outcomes.

## 1 Introduction

Breastfeeding is a cornerstone of infant nutrition and maternal health, delivering substantial physiological and psychological benefits. It provides essential nutrients and antibodies that promote cognitive development and protect against infections, obesity, and type 2 diabetes in infants (1–3). For mothers, breastfeeding is associated with a reduced risk of breast and ovarian cancers, cardiovascular disease, and type 2 diabetes, while also enhancing maternal-infant bonding (1, 2). Effective breastfeeding depends on various anatomical and physiological factors, including the integrity of breast structures responsible for milk production and transfer. Elective breast surgeries such as augmentation may alter these structures, potentially interfering with lactation (4, 5).

Silicone breast implants, favored for their natural appearance and feel, are used in over 80% of augmentation procedures in the United States (6–9). The American Society of Plastic Surgeons reported over 298,000 breast augmentation procedures in 2022, marking a continued upward trend in cosmetic surgery demand despite pandemic-related declines (10). In Saudi Arabia, shifting beauty norms and broader societal acceptance have similarly fueled a rise in cosmetic breast procedures. A national study by Alghamdi et al. (11) found that breast augmentation accounted for 31.2% of all plastic surgeries among adults in the country. Additionally, Mortada et al. (12) found that many Saudi women have limited knowledge about complications following breast implant surgery, suggesting a gap in informed decision-making.

While breast implants are generally considered safe, concerns persist regarding their impact on lactation. Surgical trauma may disrupt nerve pathways or damage milk ducts, leading to pain, latching difficulties, reduced milk supply, or early cessation of breastfeeding (13, 14). Systematic reviews have shown that women with implants have a significantly lower likelihood of exclusively breastfeeding and report higher rates of complications such as mastitis and lactation failure (15, 16). A prospective cohort study further confirmed that although many women with implants initiate breastfeeding, exclusivity rates remain significantly lower compared to women without implants (17).

Despite the increasing number of implant procedures in Saudi Arabia, region-specific studies on lactation outcomes remain scarce. Given that breastfeeding is deeply embedded in the country's cultural and religious values, this lack of data presents a critical gap in patient-centered care (18). Without access to accurate, localized information, women may struggle to make fully informed decisions about cosmetic breast surgery and its implications for future breastfeeding.

To address this gap, the present study describes breastfeeding practices and challenges among women in Saudi Arabia who have undergone breast implant surgery. Specifically, it explores breastfeeding initiation, duration, feeding method, and self-reported challenges. By examining the lived experiences of this under-researched population, the study aims to support evidence-based counseling and postpartum care tailored to the needs of women with breast implants.

## 2 Materials and methods

### 2.1 Study design

This study employed a cross-sectional descriptive design to describe lactation outcomes among women who had undergone breast augmentation. A self-administered online questionnaire was distributed once to each participant, capturing retrospective data on breastfeeding experiences up to the time of survey completion. While cross-sectional methods offer efficiency and feasibility for behavioral assessments, they do not establish causal relationships and may be subject to selection bias (19).

### 2.2 Study setting, population, and sampling

Women who had breast implants were recruited based on their willingness and availability through convenience sampling. While this method of non-probability sampling may result in selection bias, it is useful for initial research to form hypotheses and comprehension (20).

Women who met the inclusion criteria and completed the online questionnaire were included in the research. Criteria for inclusion encompassed women who were 18 years or older, had given birth within the past 1–5 years following breast implant surgery and breastfed their babies, had delivered full-term infants at an appropriate weight, and were free from neonatal illness, had no inverted nipples, tumors, or postoperative complications.

The sample size was estimated using Calculator.net, assuming a 95% confidence level, 5% margin of error, and a presumed 50% prevalence of successful breastfeeding among women with breast implants (15). With an anticipated response rate of 80%, the required sample was calculated to be 240 participants.

### 2.3 Data collection

Information was gathered through a structured survey created by the author after thoroughly reviewing various previous research studies with comparable goals (17, 21–23). The survey was created to collect detailed data across three primary categories. The initial part of the study concentrated on demographic information such as age, education, marital status, employment status, and number of children. The next part focused on the background of the participants regarding breast implants, inquiring about why they had the surgery (cosmetic, reconstructive, medical), and the type of implants used (saline, silicone, or unknown). The third part of the study looked at experiences with the most recent breastfeeding following breast implant surgery, including plans for breastfeeding before surgery, when breastfeeding started and how long it lasted, whether exclusive or mixed feeding was used, and any issues encountered like pain, inadequate milk supply, breast/nipple problems, need for extra feeding, milk production challenges, nipple sensitivity changes, impact of breast shape on baby's latch, and safety concerns about breastfeeding with implants. The survey included different ways for participants to answer, such as multiple-choice and yes/no questions to gather a broad range of responses.

Data was collected over 8 months from June 2024 to January 2025, via an Arabic online survey administered through Google Forms, accessible on both mobile and desktop devices. The survey link was disseminated using social media platforms (Facebook, Instagram, WhatsApp) and personal networks targeting women residing in Saudi Arabia.

The first page of the survey included an eligibility checklist, study details, and an informed consent statement. Participants who selected “Agree to participate” proceeded to the questionnaire; those who declined were automatically exited. Only fully completed questionnaires from participants meeting the inclusion criteria were retained for analysis. Responses from participants who did not meet the criteria or with missing data were not included. The original English version of the questionnaire was translated into Arabic and back translated to English by bilingual professionals to ensure linguistic accuracy and consistency. Content validity was confirmed by a panel of experts in pediatric nursing, obstetrics, and gynecology who evaluated each item's clarity and relevance using a 4-point scale. The Item-Level Content Validity Index (I-CVI) and Scale-Level CVI average (S-CVI/Ave) were calculated, with the final S-CVI/Ave reaching 0.90, indicating strong content validity (24). Minor revisions were made to eliminate redundancy. Internal reliability and usability of the survey were assessed through a pilot study involving 48 eligible women (20% of the total sample). Participants completed the survey and provided feedback. Based on this input, minor adjustments were made prior to full-scale data collection. The data from the pilot study was excluded from the final analysis dataset.

### 2.4 Data analysis

Data were entered, coded, and analyzed using IBM SPSS Statistics Version 29. Descriptive statistics (frequencies, percentages, means, and standard deviations) were used to summarize demographic and clinical variables. Associations between demographic factors and breastfeeding outcomes were examined using chi-square tests. Variables showing significance were further assessed using multivariable logistic regression to determine independent predictors of breastfeeding duration. Statistical significance was set at *p* < 0.05.

### 2.5 Ethical consideration

The study was conducted after being reviewed and approved by the college and the university (IRB No NRJ24/019/5, Date: 07/17/2024). Before filling out the questionnaire, all participants were given information about nature and goals of study through an online platform. At the start of the questionnaire, participants' privacy, autonomy, and confidentiality were protected by including informed consent with no names provided. All data were stored in password-protected files on secure university servers, accessible only to the principal investigator. Respondents' anonymity was protected through an online format, ensuring privacy and data integrity.

## 3 Results

The sample included 240 women aged between 23 and 33 years, with a mean age of 28.5 ± 4.11 years. Educational attainment varied: 28.3% reported no formal education, 24.2% completed high school, and 24.2% held a bachelor's degree. Regarding marital status, 39.2% were married, 32.1% divorced, and 28.8% widowed. A majority (60.4%) were employed, and participants reported between one and five children, with an average of 3.18 ± 1.41.

In terms of breast implant background, 35.4% of participants underwent cosmetic surgery, 25.0% for medical reasons, and 39.6% for reconstructive purposes. Implant type was reported as silicone by 40.4%, saline by 33.8%, and unknown by 25.8% (Table 1).

**Table 1:** Demographic characteristics and surgery details of the participants.

Variable	N	%
Age (Years)		
23–27	69	28.8
28–32	76	31.7
33	95	39.6
Number of Children		
1–2	68	28.3
3–4	117	48.8
5	55	22.9
Educational Level		
No formal education	68	28.3
High school	58	24.2
Bachelor degree	58	24.2
Postgraduate studies	56	23.3
Marital Status		
Married	94	39.2
Divorced	77	32.1
Widowed	69	28.8
Occupation		
Employed	145	60.4
Unemployed	95	39.6
Reason for Surgery		
Cosmetic	85	35.4
Medical	60	25.0
Reconstructive	95	39.6
Type of Implants		
Saline	81	33.8
Silicone	97	40.4
Unknown	62	25.8

Prior to surgery, 42.5% intended to breastfeed. Postpartum, 50.4% initiated breastfeeding immediately, while the remaining 49.6% experienced delays. Breastfeeding duration varied: 36.7% breastfed for less than one month, 35.8% for 1–6 months, and 27.5% for more than 6 months. Exclusive breastfeeding was reported by 46.3%, while 53.8% practiced mixed feeding.

Reported breastfeeding challenges included pain during feeding (55.0%), perceived insufficient milk supply (48.3%), and breast or nipple issues (48.3%). Other concerns included milk production difficulties (33.3%), nipple sensitivity changes (4.2%), and the use of supplementary feeding (49.6%). A subset of participants reported latching difficulties attributed to altered breast shape (20.8%) and safety concerns about breastfeeding with implants (16.7%) (Table 2).

**Table 2:** Breastfeeding intentions, experience, and problems among the participants.

Variables	N	%
Intentions to breastfeed before surgery		
Yes	102	42.5
No	138	57.5
Start of breastfeeding		
Immediately after birth	121	50.4
Delayed	119	49.6
Duration of breastfeeding		
Less than 1 month	88	36.7
1–6 months	86	35.8
More than 6 months	66	27.5
Breastfeeding method		
Exclusive	111	46.3
Mixed	129	53.8
Pain during breastfeeding		
Yes	132	55.0
No	108	45.0
Perceived insufficient milk supply		
Yes	116	48.3
No	124	51.7
Breast or nipple issues		
Yes	116	48.3
No	124	51.7
Need for supplementary feeding		
Yes	119	49.6
No	121	50.4
Difficulties with milk production		
Yes	80	33.3
No	160	66.7
Changes in nipple sensitivity affecting breastfeeding		
Yes	10	4.2
No	230	95.8
Impact of breast shape post-surgery on baby's latch		
Yes	50	20.8
No	190	79.2
Safety concerns regarding breastfeeding with implants		
Yes	40	16.7
No	200	83.3

Bivariate analysis revealed statistically significant associations between breastfeeding duration and maternal age (*p* = .041), marital status (*p* = .027), and number of children (*p* = .026). In the multivariable analysis, only marital status remained a significant predictor of breastfeeding duration: married women had lower odds of breastfeeding beyond six months compared to divorced women (OR=0.38, 95% CI: 0.17–0.87, *p* = 0.022). No significant associations were observed between breastfeeding outcomes and education level or employment status (Table 3).

**Table 3:** Duration of breastfeeding by demographic characteristics of the participants.

Variable	1–6 months	Less than 1 month	More than 6 months	*P*-value
Age				.041*
23–27	24 (10.0%)	27 (11.3%)	18 (7.5%)	
28–32	28 (11.7%)	29 (12.1%)	19 (7.9%)	
33 and above	34 (14.2%)	32 (13.3%)	29 (12.1%)	
Number of children				.026*
1–2	18 (7.5%)	27 (11.3%)	23 (9.6%)	
3–4	47 (19.6%)	35 (14.6%)	35 (14.6%)	
5	21 (8.7%)	26 (10.8%)	8 (3.3%)	
Educational Level				.546
No formal education	31 (12.9%)	22 (9.2%)	15 (6.2%)	
High school	18 (7.5%)	24 (10.0%)	16 (6.7%)	
Bachelor degree	20 (8.3%)	22 (9.2%)	16 (6.7%)	
Postgraduate studies	17 (7.1%)	20 (8.3%)	19 (7.9%)	
Marital Status				.027*
Married	35 (14.6%)	42 (17.5%)	17 (7.1%)	
Divorced	32 (13.3%)	22 (9.2%)	23 (9.6%)	
Widowed	19 (7.9%)	24 (10.0%)	26 (10.8%)	
Occupation				.475
Employed	50 (20.8%)	51 (21.3%)	44 (18.3%)	
Unemployed	36 (15.0%)	37 (15.4%)	22 (9.2%)	

* Significant difference.

## 4 Discussion

The research depicted a diverse demographic background of the participants. The average age was 28.5 years, which falls into the normal trend of studies involving breast implants that have patients in their mid−20s and early 30s (17, 22). The educational status distribution in this study showed that nearly a quarter of participants had no formal education, with the remaining participants having completed high school, bachelor's, or postgraduate degrees. In contrast, it was found that the majority of women undergoing breast implant surgeries in Saudi Arabia were well-educated, with most holding at least a bachelor's degree, while only a small fraction had no formal education (12). The reason for surgery in this study was cosmetic and reconstruction for three-quarters of the participants. In comparison, it was reported that 58.5% of women underwent breast implant surgery for reconstructive purposes and the remaining 41.5% for cosmetic reasons (12). This may suggest that demographic differences can influence the motivations for breast implants. In this study the most commonly used implants were the silicone ones, indicating an observed preference across many studies. This trend is likely attributed to the more natural feel and texture of silicone implants compared to saline alternatives (6, 17, 23).

In terms of lactation outcomes, 42.5% of participants reported planning to breastfeed prior to delivery, and 50.4% initiated breastfeeding immediately postpartum. Exclusive breastfeeding was reported by 46.3% of the sample, while mixed feeding was slightly more common at 53.8%. These findings are consistent with prior research suggesting that mixed feeding practices may be prevalent among women with a history of breast surgery, although differences across studies in design, population, and definitions of exclusivity warrant caution in direct comparisons (15, 21).

Broadly, breastfeeding among Saudi mothers showed variable trends. According to a national survey in 2023, the rate of exclusive breastfeeding at six months was 15% (25). In Taif, exclusive breastfeeding prevalence was 16.3% among infants aged 6–12 months (26), while a study in Tabuk found 31.4% of mothers exclusively breastfed to six months (27).

Pain during breastfeeding was reported by 55% of participants, while 48.3% cited perceived insufficient milk supply. These difficulties mirror challenges frequently documented in postpartum populations more broadly but have also been observed in previous studies involving women with breast implants. Some earlier reports have documented lower rates of breastfeeding pain than seen in this study (22). Additionally, nearly half of participants (49.6%) reported using supplementary feeding, further underscoring the complexity of breastfeeding experiences in this group and the potential influence of both physical and psychosocial factors.

Maternal age, marital status, and number of children were significantly associated with breastfeeding duration in the bivariate analysis. However, logistic regression model revealed only marital status statistically significant. Married women had 62% lower odds of breastfeeding beyond six months compared to divorced women (OR = 0.38, 95% CI: 0.17–0.87, *p* = 0.022). This finding contrasts with prior research (28, 29) where partner support was associated with longer breastfeeding duration. Possible explanation is that divorced women in this sample may have possessed more flexible daily schedules, which could facilitate sustained breastfeeding.

The number of children was also significantly associated with breastfeeding duration, suggesting that maternal experience may play a key role in sustaining breastfeeding. In this study, multiparous women reported longer breastfeeding durations compared to first-time mothers, consistent with prior research indicating that prior experience can enhance maternal confidence and persistence (30). However, no significant associations were found between number of children and breastfeeding intention or feeding method. This pattern implies that while previous parenting experience may not strongly shape prenatal decisions about breastfeeding, it may contribute to greater resilience and ability to continue breastfeeding despite potential challenges.

No significant associations were observed between educational level and any breastfeeding outcomes in this study. While this may appear inconsistent with international findings that often link higher education to improved breastfeeding practices, it aligns with research specific to the Saudi Arabian context. In this setting, women with lower levels of formal education may be more likely to adhere to traditional cultural norms and familial expectations that strongly favor breastfeeding, potentially offsetting the influence of formal education on breastfeeding behaviors (31).

This study addresses a gap in regional research by examining patterns of breastfeeding among women with breast implants in Saudi Arabia. By gathering detailed data on breastfeeding initiation, duration, methods, and self-reported challenges, the study provides considerable insights into the experiences of this group, potentially informing culturally sensitive clinical guidance and postpartum care practices. The use of a cross-sectional design enabled efficient data collection from a relatively large sample, supporting broad descriptive analysis within the target demographic.

However, limitations of the study include the use of convenience sampling, which may introduce selection bias as well as the inability to establish causation due to the cross-sectional nature of the study. Additionally, self-reported data may be subject to recall bias. Another limitation is the absence of a control group consisting of women who have not undergone any breast surgery.

To support informed decision-making among women considering breast implant surgery, healthcare providers should incorporate preoperative counseling that addresses potential implications for breastfeeding. Clear, evidence-based discussions about lactation outcomes can help align patient expectations with surgical decisions. Postpartum care should also be tailored to the needs of women with breast implants, offering targeted lactation support that addresses common challenges such as latching difficulties and concerns about milk production. Developing culturally sensitive breastfeeding education programs that specifically account for anatomical changes and patient concerns related to implants may further enhance breastfeeding success. Future research is needed to examine the long-term effects of breast implants on lactation, particularly within the sociocultural context of Saudi Arabia, where traditional values and family structures may influence breastfeeding behaviors and health-seeking decisions.

## 5 Conclusion

This study highlights that breastfeeding is feasible for many women in Saudi Arabia with breast implants, although various challenges are frequently reported. Approximately half of the participants initiated breastfeeding postpartum, but continuation beyond six months was less common, and mixed feeding was more prevalent than exclusive breastfeeding. In adjusted analysis, marital status was the only demographic factor significantly associated with breastfeeding duration. Commonly cited concerns included pain, perceived insufficient milk supply, and breast or nipple discomfort, while issues such as altered nipple sensitivity, latching difficulties related to breast shape, and safety concerns about breastfeeding with implants were reported less frequently.

## Data Availability

The raw data supporting the conclusions of this article will be made available by the authors, without undue reservation.
