# Supplementary material for: Breastfeeding practices and challenges in women with breast implants in Saudi Arabia: a descriptive study
Source: Front Glob Womens Health. 2025 Sep 2;6:1647351. doi: 10.3389/fgwh.2025.1647351 (PMC12436290; doi:10.3389/fgwh.2025.1647351)
Supplement: Supplementary file 1 [file Table1.docx]

Supplementary Material

**Supplementary Table 1.** Intentions to breastfeed by demographic characteristics of the participants:

| Variable | No | Yes | *P*-value |
| --- | --- | --- | --- |
| Age |  |  | .994 |
| 23-27 | 41 (17.1%) | 28 (11.7%) |  |
| 28-32 | 41 (17.1%) | 35 (14.6%) |  |
| 33 | 56 (23.3%) | 39 (16.2%) |  |
| Number of children |  |  | .915 |
| 1-2 | 38 (15.8%) | 30 (12.5%) |  |
| 3-4 | 70 (29.2%) | 47 (19.6%) |  |
| 5 | 30 (12.5%) | 25 (10.4%) |  |
| Educational Level |  |  | .248 |
| No formal education | 33 (13.8%) | 35 (14.6%) |  |
| High school | 35 (14.6%) | 23 (9.6%) |  |
| Bachelor degree | 33 (13.8%) | 25 (10.4%) |  |
| Postgraduate studies | 37 (15.4%) | 19 (7.9%) |  |
| Marital Status |  |  | .552 |
| Married | 58 (24.2%) | 36 (15.0%) |  |
| Divorced | 43 (17.9%) | 34 (14.2%) |  |
| Widowed | 37 (15.4%) | 32 (13.3%) |  |
| Occupation |  |  | >.999 |
| Employed | 83 (34.6%) | 62 (25.8%) |  |
| Unemployed | 55 (22.9%) | 40 (16.7%) |  |

**Supplementary Table 2.** Start of breastfeeding by demographic characteristics of the participants:

| Variable | Delayed | Immediately after birth | *P*-value |
| --- | --- | --- | --- |
| Age |  |  | .767 |
| 23-27 | 38 (15.8%) | 31 (12.9%) |  |
| 28-32 | 32 (13.3%) | 44 (18.3%) |  |
| 33 | 49 (20.4%) | 46 (19.2%) |  |
| Number of children |  |  | .842 |
| 1-2 | 31 (12.9%) | 37 (15.4%) |  |
| 3-4 | 62 (25.8%) | 55 (22.9%) |  |
| 5 | 26 (10.8%) | 29 (12.1%) |  |
| Educational Level |  |  | .474 |
| No formal education | 32 (13.3%) | 36 (15.0%) |  |
| High school | 34 (14.2%) | 24 (10.0%) |  |
| Bachelor degree | 27 (11.3%) | 31 (12.9%) |  |
| Postgraduate studies | 26 (10.8%) | 30 (12.5%) |  |
| Marital Status |  |  | .655 |
| Married | 50 (20.8%) | 44 (18.3%) |  |
| Divorced | 37 (15.4%) | 40 (16.7%) |  |
| Widowed | 32 (13.3%) | 37 (15.4%) |  |
| Occupation |  |  | .139 |
| Employed | 78 (32.5%) | 67 (27.9%) |  |
| Unemployed | 41 (17.1%) | 54 (22.5%) |  |
